# ^18^F-FDG PET/CT imaging in pulmonary sarcomatoid carcinoma

**DOI:** 10.3389/fonc.2024.1334156

**Published:** 2024-02-14

**Authors:** Zhe-Huang Luo, Xiao-Yan Luo, Wan-Ling Qi, Qian Liu

**Affiliations:** ^1^ Department of Nuclear Medicine, Jiangxi Provincial People’s Hospital, The First Affiliated Hospital of Nanchang Medical College, Nanchang, China; ^2^ Department of Clinical Laboratory, Jiangxi Provincial Children’s Hospital, The Affiliated Children’s Hospital of Nanchang Medical College, Nanchang, China; ^3^ Department of Pathology, Jiangxi Provincial People’s Hospital, The First Affiliated Hospital of Nanchang Medical College, Nanchang, China

**Keywords:** pulmonary sarcomatoid carcinoma, fluorodeoxyglucose, positron emission tomography/computed tomography, imaging features, TTF-1 expression

## Abstract

**Background:**

Pulmonary sarcomatoid carcinoma (PSC) is a rare highly aggressive and poorly differentiated non-small cell carcinoma, and little is known about the information on the usefulness of ^18^F-fluorodeoxyglucose (^18^F-FDG) positron emission tomography/computed tomography (PET/CT). We investigated the clinical and ^18^F-FDG PET/CT features of PSC.

**Methods:**

We retrospectively analyzed 25 consecutive PSC patients who had undergone ^18^F-FDG PET/CT. Demographic data, PET/CT findings before treatment, pathological features, and prognosis in these patients were investigated to define correlates between maximal standard uptake value (SUVmax) and clinicopathological parameters.

**Results:**

From March 2017 to January 2023, twenty-five eligible patients with PSC were identified. There were 23 (92%) men, aged 68.5 ± 8.5 (range 56-90) years. Eighteen (72%) patients had a frequent smoking history. The mean size of PSCs was 59.3 ± 18.6 (range 29-97) mm, and 23 (92%) PSCs were Stage IV tumors. 20 (80%) lesions were located in the upper lung and 19 (76%) cases belonged to the peripheral type. Necrotic foci appeared in 21(84%) tumors. 11 (44%) PSCs invaded the pleura. All PSCs were FDG avid, and the mean of SUVmax was 11.8 ± 5.3 (range 4.8-25.5). Metastases were found on PET/CT in 24(96%) patients. The SUVmax of the lesions ≥ 5cm was higher than that of the lesions < 5cm (p=0.004), and the SUVmax of lesions with TTF-1 expression was higher than those of lesions without TTF-1 expression (p=0.009). All of the 25 primary lesions were considered malignant and confirmative, probable, and possible diagnosis of PSC was made in 2 (8%), 4 (16%), and 5(20%) patients, respectively on PET/CT. PSC was not considered in 14 (56%) patients, in PET/CT. The survival of patients with surgery didn’t demonstrate a significantly good prognosis as compared with those without surgery (p=0.675).

**Conclusion:**

All PSCs had obvious FDG avidity. Although imaging diagnosis is still difficult, combined clinical and imaging features more than 40% of primary lesions were considered for the possibility of PSC in our group. Early histopathological diagnosis is necessary to help develop a reasonable regimen.

## Introduction

Sarcomatoid carcinoma (SC) is a rare highly aggressive and poorly differentiated malignant tumor, which is characterized by a combination of malignant epithelial and mesenchymal cells. SC can occur in various organs such as the lungs, liver, kidney, breast, and bladder. The lung was the most common location in which SC occurs. Pulmonary SC(PSC) is an unusual type of non-small-cell lung cancer (NSCLC) and accounts for 0.1% to 0.4% of total NSCLC (1). PSC consists of 5 pathological subtypes according to the 2015 WHO (World Health Organization) classification system: pleomorphic carcinoma, spindle cell carcinoma, giant cell carcinoma, carcinosarcoma, and pulmonary blastoma. Because PSC is highly malignant and insensitive to chemoradiotherapy, the prognosis is dismal (2).

Due to its rarity and heterogeneity, PSC is usually misdiagnosed. However, given its high malignancy and very poor prognosis, it is essential to obtain a definite diagnosis. The importance of imaging modalities has been highlighted to improve the diagnosis of pulmonary diseases (3). ^18^F-FDG PET/CT has been widely used in the diagnosis, staging, surveillance, and prognostication of various tumors, including malignancies in the lung. However, very few coherent studies have investigated the potential role of ^18^F-FDG PET/CT in the diagnosis of PSC, with most case studies (4–7). A few studies (8, 9) have conducted a detailed study of ^18^F-FDG PET/CT imaging in PSC patients, but have reached inconsistent conclusions. In the present study, we retrospectively evaluated ^18^F-FDG PET/CT findings of 25 patients with PSC and analyzed the possible correlates of PET/CT parameters with the other features of the PSCs.

## Materials and methods

### Ethical standards and patient inclusion criteria

This is a single-center, retrospective study with clinical data and ^18^F-FDG PET/CT parameters from the electronic medical records. The study was performed under the ethical standards laid down in the 1964 Declaration of Helsinki and its later amendments. The study was submitted to the Institutional Ethics Committee of Jiangxi Provincial People’s Hospital and was deemed exempt from ethical review. Written informed consent was obtained from the individuals or next of kin for the publication of any potentially identifiable images or data included in this article.

Patient inclusion criteria: 1. PSC was histopathologically confirmed; 2. From March 2017 to March 2023, the patient has undergone an ^18^F-FDG PET/CT examination in the PET/CT center, at Jiangxi Provincial People’s Hospital; 3. There’s only one primary lesion. 4. No systemic or local antitumor therapy was received before PET/CT examination; 5. there wasn’t a history of malignancy or no other coexisting malignant disorder; 6. Complete case records were available.

### ^18^F-FDG PET/CT imaging

All patients were instructed to fast for at least 6 h and their blood glucose levels were kept lower than 7.4 mmol/l. Image acquisition was taken 45~60 min after intravenous administration of 5.6 MBq of ^18^F-FDG per kilogram of body weight. All scans were performed on a GE Healthcare Discovery STE system. First, a CT scan from the vertex of the skull to the upper thigh with the patient supine was performed, under the following conditions: 120KV, auto mA, pitch 1.75:1; collimation 16×3.75 mm; and rotation cycle, 0.5 s, for PET attenuation correction and anatomical location, without oral or intravenous contrast. Then, PET images covering the same area were acquired in three‐dimensional mode on the same scanner for 2.5 min per bed position, a total of 6-7 bed positions per patient. PET images were reconstructed using CT for attenuation correction with the ordered-subsets expectation maximization algorithm (2 iterations, 28 subsets) and an 8-mm gaussian filter using a 128×128 matrix. All images were reviewed on a picture-archiving and communication system (PACS) workstation (GE AW 4.6 workstation) displaying a maximum-intensity projection image (MIP) and multiplanar PET, CT, and PET/CT fusion images.

### Data analysis

The diagnosis of PSC is based on the morphological characteristics of tumor cells and immunohistochemical staining. The clinical data of the eligible patients were collected from the electronic medical records, including demographic data, smoking history, and serum tumor markers associated with lung cancer such as CEA (carcinoembryonic antigen), SCCA (squamous cell carcinoma antigen), NSE (neuron-specific enolase), and CYFRA21-1 (cytokeratin 21-1).

^18^F-FDG PET/CT of all patients were retrieved. Imaging data were reinterpreted by two board-certified nuclear medicine physicians who didn’t know patients’ clinical information, other conventional imaging findings, and pathology results. All images were analyzed visually and semi-quantitatively with the measurement of the standardized uptake value (SUV). The SUV values were generated utilizing the patient’s body mass index. The SUV of the lesions was measured by placing the cursor at ROI (region of interest) and one-click measurement of workstation AW4.6. PET/CT parameters were assessed as follows: tumor size, lobulation, pleura invasion, necrosis, metastasis, and maximum standardized uptake value (SUVmax).

According to the clinical features and frequent imaging findings of PSC (4–9), we applied a tentative diagnostic criterion of PET/CT imaging for PSM: 1)Male, 2) ≥ 60 years old, 3) long smoking history, 4) a lobulated mass, 5) located in the upper lobe, 6) the maximum diameter > 5cm, 7) broad pleural invasion, 8) without definite calcification, 9)Large intrinsic herterogeneous necrotic area, 10) hypermetabolism in the solid component (a, SUVmax ≥ 15; b, 8≤ SUVmax <15; c, SUVmax <8). Diagnosis: 1-9 and 10a; probable diagnosis: 2 + 4 + 7+9 + 10ab; possible diagnosis: 4 + 9 + 10abc. The two doctors recorded these parameters and gave a preliminary diagnosis. If there is a disagreement, it will be settled through negotiation. A correct diagnosis of type is defined as a definitive diagnosis or a probable diagnosis consistent with the pathology.

### Follow-up

After PET/CT examinations, all patients underwent routine follow-ups, which included evaluation of symptoms, laboratory test results, imaging findings, and pathological results. The follow-up methods included outpatient examination, telephone or web chat follow-up, and inpatient medical records. The final follow-up was completed by the end of March 2023. Overall survival (OS) was defined as the time from the initial diagnosis to the patient’s death.

### Statistical analysis

SPSS 19.0 software for Windows (IBM Corp., Armonk, NY, USA) was used for the statistical analysis. Continuous variables are expressed as mean ± standard deviation (SD) or median, and categorical variables are presented as numbers and percentages (%). Correlates between SUVmax and the other image characteristics were compared and studied by the independent sample t-test. P values < 0.05 were considered significant. Overall survival (OS) was assessed using the Kaplan–Meier method and univariate analyses were performed using the log-rank test.

## Results

### Demographic data and clinical characteristics

**Figure 1:**
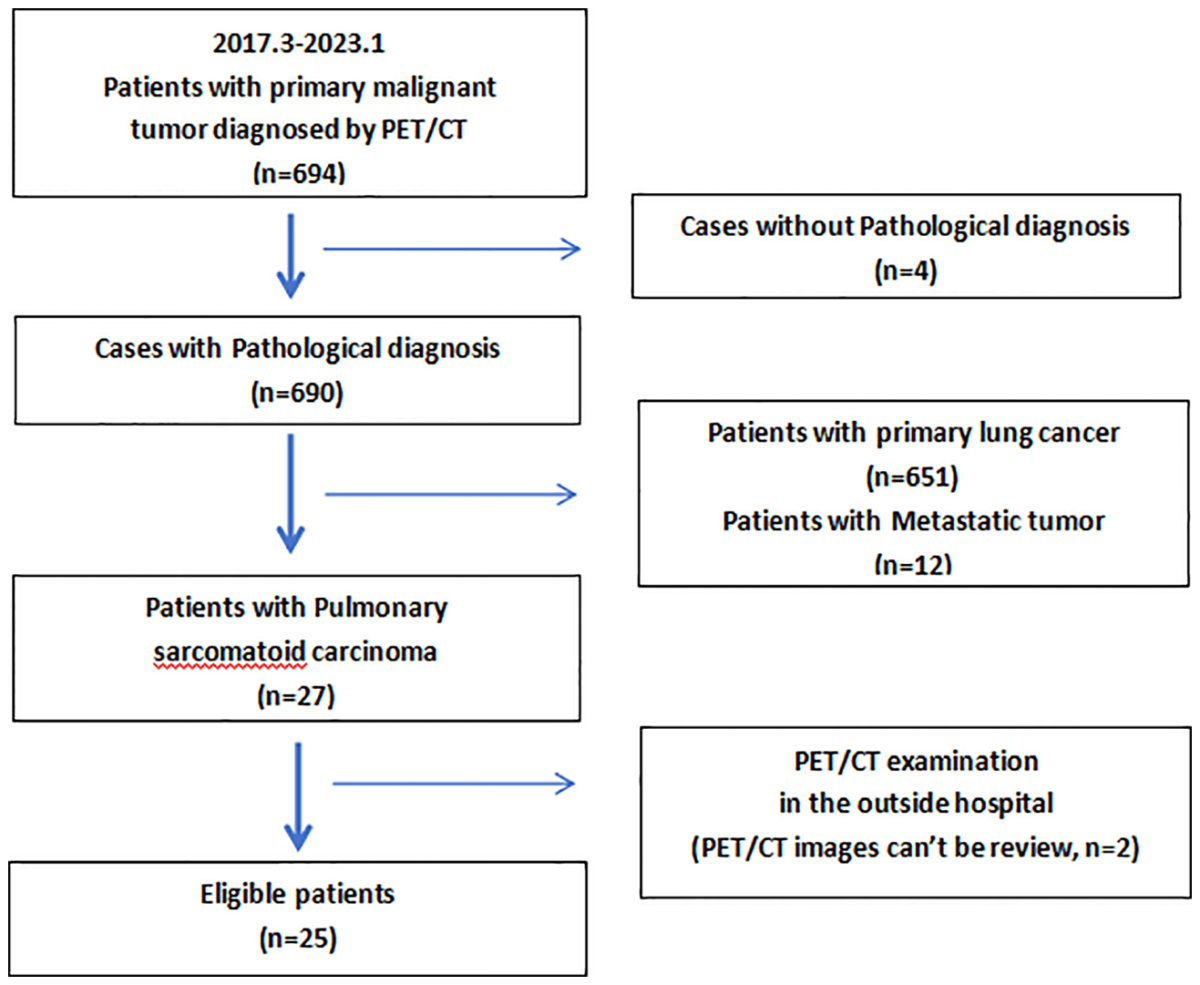
Flowchart of the eligible patients.

A total of 694 patients who had undergone PET/CT before treatment at Jiangxi Provincial People’s Hospital from March 2017 to January 2023 were diagnosed histopathologically with primary pulmonary malignancy in which 25 eligible patients with PSC were identified (**Figure 1**). Of the 25 patients, there were 23 men and 2 women aged 68.5 ± 8.5(range 56-90) years. Seventy-two percent (18/25) patients had a frequent smoking history (≥5 packs/month, 20 cigarettes/pack), and the remaining 7(28%) patients (including the two women) denied ever smoking. All patients had a single primary lesion. Cough was the most common symptom. According to the eighth edition of the TNM staging system of lung cancer edited by the Union for International Cancer Control (UICC) in 2017, 92% (23/25) patients had stage IV tumors. The Demographic data, clinical characteristics, and serum tumor marker levels associated with PSC were summarized in **Table 1**. Five (20%) patients received surgical treatment and the other 20(80%) patients received non-surgical treatment.

**Table 1:** Demographic data and clinical characteristics.

Clinical features	Pulmonary Sarcomatoid Carcinoma (n=25)
Gender, n (%)	
** Male**	23(92.0%)
** Female**	2(8.0%)
Age, (years)	
** mean**	68.6 ± 8.5
** range**	56-90
Smoking history, n (%)	
** frequently**	18(72.0%)
** never**	7(28.0%)
Symptoms, n (%)	
** Cough**	18(72.0%)
** Hemoptysis**	4(16.0%)
** Chest pain**	7(28.0%)
** Fever**	3(12.0%)
Primary sites, n (%)	
** Left upper lobe**	10(40.0%)
** Right upper lobe**	10(40.0%)
** Left lower lobe**	2(8.0%)
** right middle lobe**	1(4.0%)
** Right lower lobe**	2(8.0%)
Stage	
** IIa+IIb**	2(8.0%)
** IVa**	16(64.0%)
** IVb**	7(28.0%)
Metastases, n (%)	
** Yes**	23(92.0%)
** No**	2(8.0%)
Metastatic sites, n (%)	
** Pleura**	11(44.0)
** Lymph node**	15(60.0)
** Lung**	8(32.0)
** Bone**	7(28.0)
** Brain**	3(12.0)
** Muscle**	2(8.0)
** Small intestine**	1(4.0)
Patients with elevated tumor markers level, n(%)	
** CEA**	3 (12.0%)
** SCC**	6 (24.0%)
** CYFRA21-1**	9 (36.0%)
** NSE**	9 (36.0%)

### ^18^F-FDG PET/CT finding

**Figure 2:**
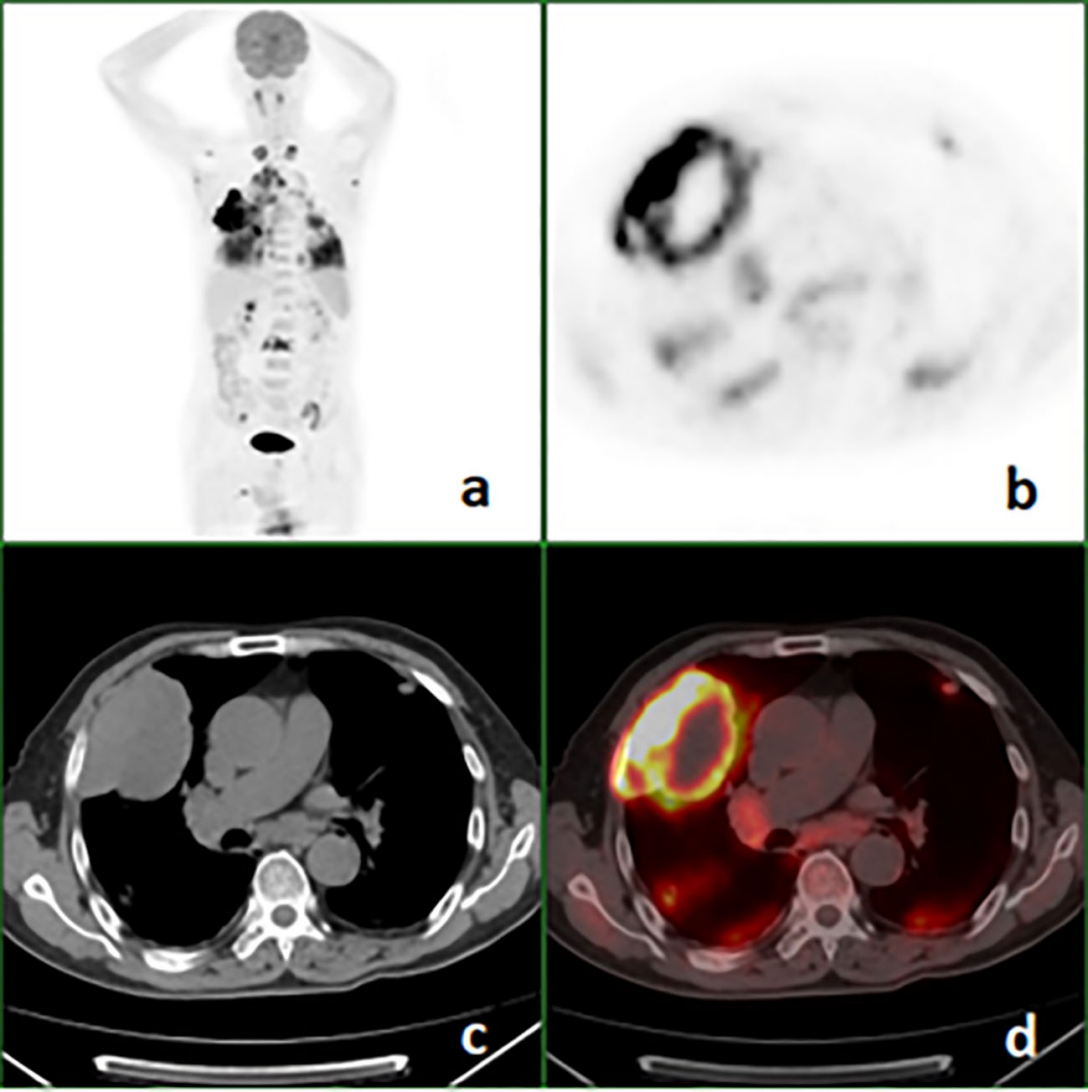
^18^F-FDG PET/CT images of a 68-year-old man with pulmonary sarcomatoid carcinoma showing a large mass in the right upper lung with broad pleural invasion, Lung and bone metastases. **(A)** PET maximal intensity projection (MIP); **(B)**, PET; **(C)**, axial CT (mediastinal window); **(D)**, fusion image.

**Figure 3:**
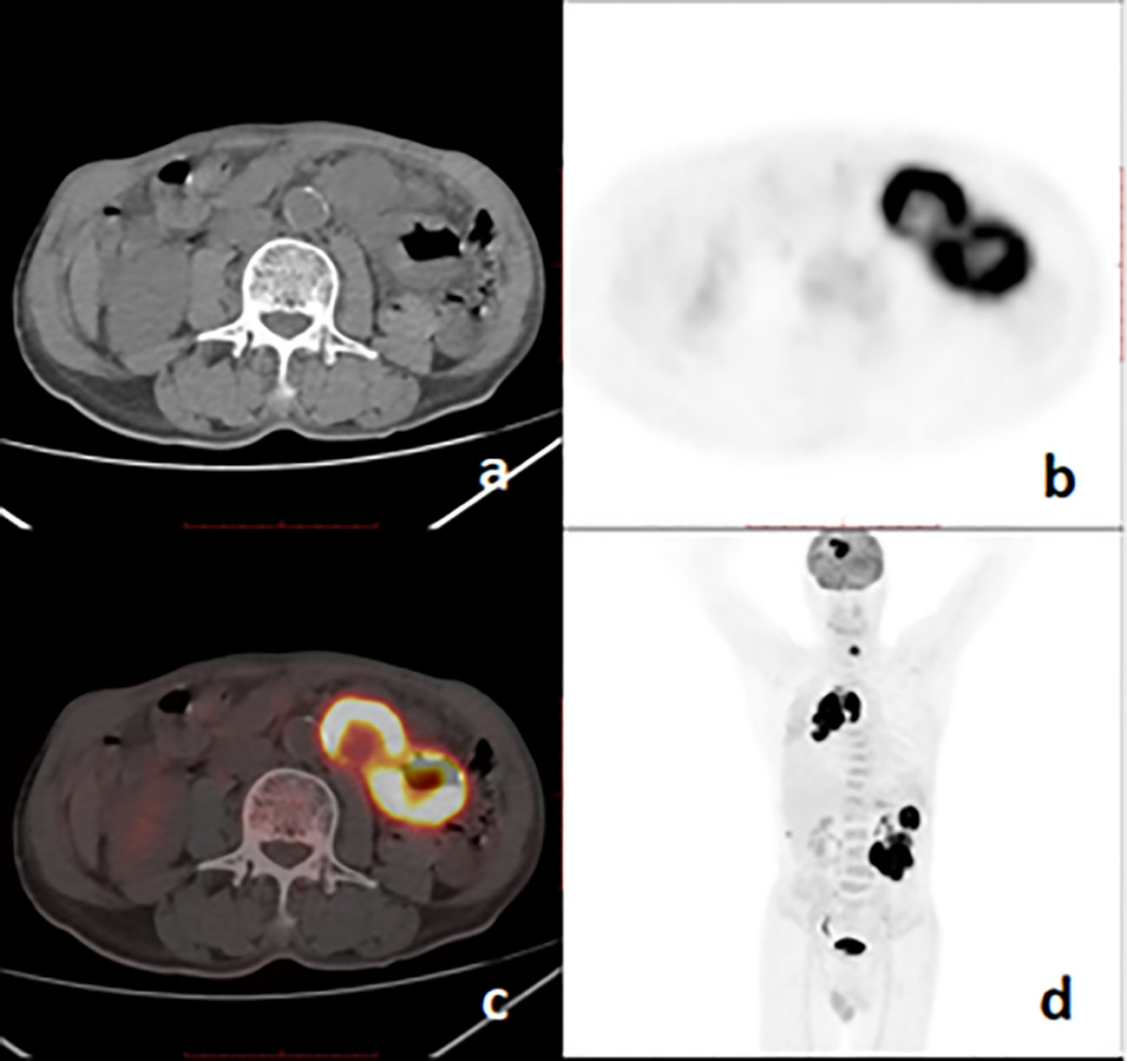
^18^F-FDG PET/CT images of a 67-year-old man with pulmonary sarcomatoid carcinoma showing a large mass with brain, bone, and intestinal metastases. **(A)**, axial CT; **(B)**, PET; **(C)**, fusion image; **(D)**, PET MIP.

Among 25 patients, PSCs were located in the right or left upper lung lobes in 20 (80%) cases, and 19 (76%) cases belonged to the peripheral type. Except for 1 (4%) tumor, the other 24 (96%) tumors were larger than 30mm in the major axis, the mean size was 59.3 ± 18.6 (range 29-97) mm. Necrotic foci of different size appeared in 21(84%) tumors. Eleven (44%) patients with invasion of the pleural abutting the tumor, nine (36%) had broad pleura invasion (**Figure 2**). Fourteen (56%) primary lesions were irregular in shape. All primary tumors were FDG avid, and the average value of SUVmax of primary lesions was 11.8 ± 5.3 (range 4.8-25.5). Metastases were found on PET/CT in 24(96%) patients (**Figure 3**), and the locations of metastases were shown in **Table 1**, Lymph node metastasis and pleural invasion were common. Among the 23 stage IV lesions, the SUVmax of 16 lesions with stage IVa and 7 lesions with stage IVb was 11.0 ± 4.1 and 15.4 ± 6.6, respectively.

### The preliminary diagnosis on PET/CT and the final pathological diagnosis

All of the 25 primary lesions were considered malignant in PET/CT; a definitive diagnosis of PSC was made in 2 (8%) patients, probable diagnosis in 4 (16%) patients, possible diagnosis in 5(20%) patients, and incorrect diagnosis in 14(56%) patients. The accuracy of qualitative diagnosis reached 100%. Correct diagnosis (definitive and probable) of PSC was made in 6 (24%) cases, and PSC was suspected (possible) in 5(20%) cases. In addition, PET/CT scans of another 308 non-PSC patients were also reviewed, a definitive diagnosis, a probable diagnosis, and a possible diagnosis of PSC were made on PET/CT in 2 (8%) patients, in 3 (10) patients, and 5 (20%) patients, respectively.

The final pathologic diagnosis was made by surgical resection (n=5), transbronchial lung biopsy (n=3), or transthoracic biopsy (n=17). Histologically, the tumors included 9 pleomorphic carcinomas, 2 spindle cell carcinomas, and 14 PSCs without subtype classification. Immunohistochemical staining of cell block sections was performed for Thyroid Transcription Factor 1 (TTF-1) in 14 cases, which showed nuclear positivity in 9 cases.

### Correlates between SUVmax and the other image characteristics (**Table 2**)

**Figure 4:**
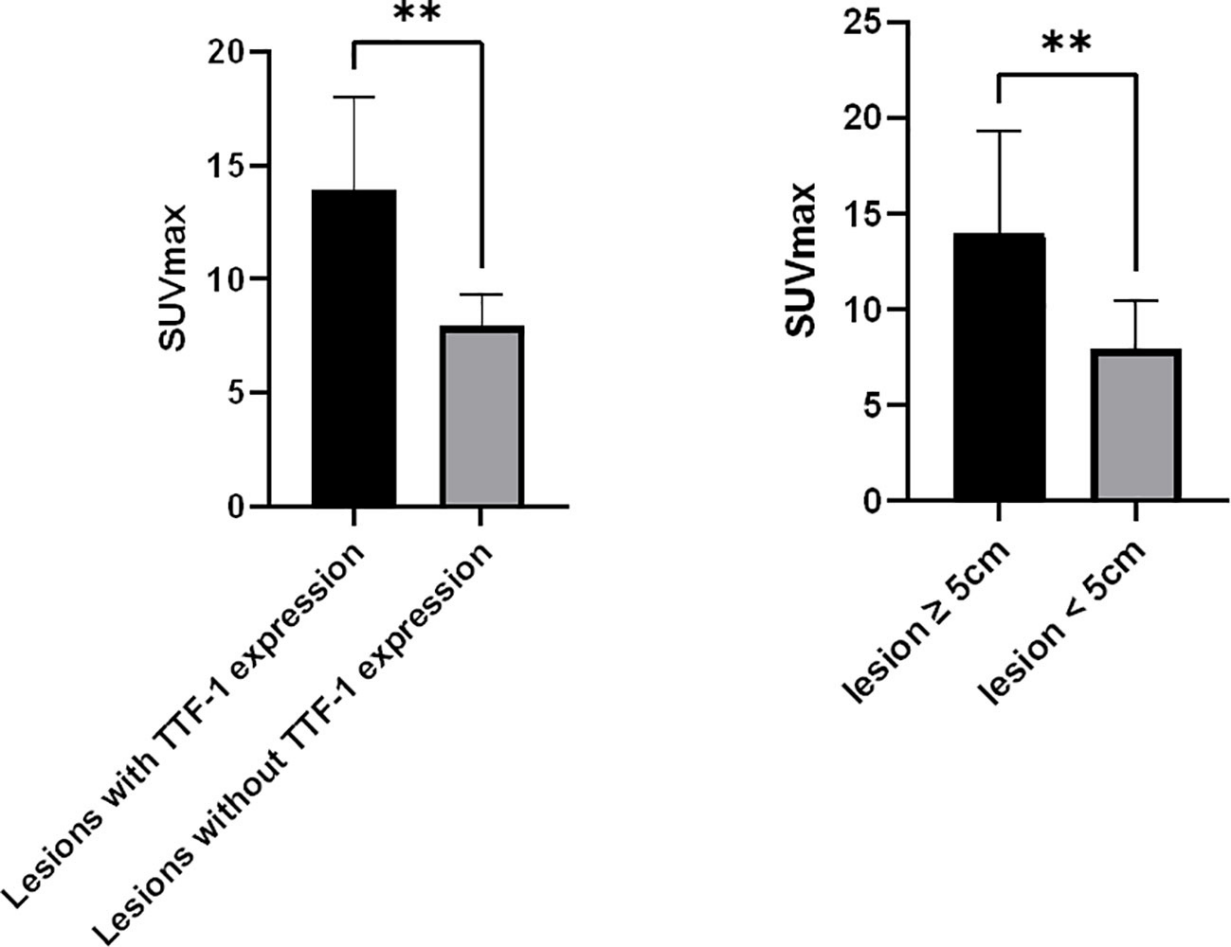
Correlates between SUVmax of PSC and tumor TTF-1 expression, Tumor size. **(A)**, SUVmax of lesions with TTF-1 expression (n=9) was higher than that of lesions without TTF-1 expression (n=5); **(B)**, SUVmax of lesions ≥ 5cm (n=16) was higher than that of lesions < 5 cm (n=9). **p < 0.01.

Of the 25 primary lesions, the longest diameter was ≥ 5cm in 19 cases and < 5cm in 6 cases, and the mean of the SUVmax was 14.0 ± 5.3 and 8.0 ± 2.5, respectively, there was a significant difference between the ≥ 5cm group and < 5cm group (p=0.004, 95% confidence interval (CI) 2.123 - 9.940), the SUVmax of the ≥ 5cm lesions was higher than that of the < 5cm lesions. The SUVmax showed no significant difference between lesions with pleural invasion (12.2 ± 5.3) and those without pleural invasion (11.5 ± 5.6) (p=0.774, CI -3.895 - 5.169), between lesions with necrosis (12.4 ± 5.5) and those without necrosis(9.7 ± 4.2) (p=0.327, CI -2.846 - 8.186), between peripheral lesions (10.8 ± 4.6) and central lesions (15.0 ± 6.7)(p=0.092, CI -0.743 - 9.166), between IVa (11.0 ± 4.1) and IVb (15.4 ± 6.5) PSCs (p =0.063, CI -0.2577 - 9.0167), and between cases with an OS ≥ 12 months(10.3 ± 5.9 and cases with an OS < 12 months (12.5 ± 5.1) (p=0.332, CI -6.997 - 2.4655). Among the 14 TTF-1 stained lesions, there was a significant difference in SUVmax between 9 positive lesions and 5 negative lesions (13.9 ± 4.1 vs 8.0 ± 1.3, p=0.009, CI 1.7802 - 10.1042) (**Figure 4**). This study also analyzed the correlation between MTV, TLG and survival, but the results showed that there was no significant correlation between them and survival time.

**Table 2:** Correlates between SUVmax and the other image characteristics.

	SUVmax	p-value
**Size (cm)**		0.004
≥5 (n=16)	14.0 ± 5.3	
<5 (n=9)	8.0 ± 2.5	
**Pleural invasion**		0.774
Yes (n=11)	12.2 ± 5.3	
No (n=15)	11.5 ± 5.6	
**Necrosis**		0.327
Yes (n=20)	12.4 ± 5.5	
No (n=5)	9.7 ± 4.2	
**Location**		0.092
Peripheral (p=19)	10.8 ± 4.6	
Central (n=6)	15.0 ± 6.7	
**Stage**		0.063
IVa (n=16)	11.0 ± 4.1	
IVb (n=7)	15.4 ± 6.6	
**OS**		0.332
≥ 12 months (n=8)	10.3 ± 5.9	
< 12 months (n=17)	12.5 ± 5.1	

OS, Overall survival.

### Follow-up and clinical outcome

**Figure 5:**
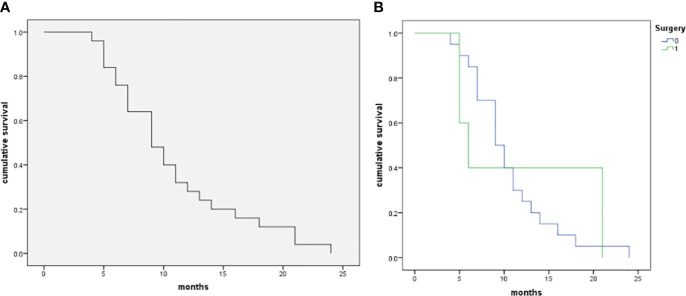
The Kaplan–Meier curve of patients with pulmonary sarcomatoid carcinoma in the patient cohort (n=25). **(A)**, The median OS of the 25 patients with PSCs was 9 months, and 12-month and 24-month OS rates were 32.0% and 0.0%, respectively. **(B)**, Comparison of survival rate between operative and non-operative patients (log-rank test, p=0.675).

The follow-up period was 11.9 ± 5.8 (range 6-24) months. During follow-up periods, all patients died of PSC-related events. There were 8 cases with an OS ≥ 12 months and 17 cases with an OS < 12 months. The overall survival curve in the five patients with surgery and 20 patients without surgery is shown in **Figure 5**. The survival of patients with surgery didn’t demonstrate a significantly good prognosis as compared with those without surgery (p=0.675).

## Discussion

PSC is a group of rare disorders. In the present study, we investigated the clinical information and PET/CT features of 25 patients with PSC, and the correlates between SUVmax of PSC and other characteristics of PSC were evaluated. To the best of our knowledge, this is one of the ^18^F-FDG PET/CT study with a larger PSC sample. We had some similar findings to previous investigations (4, 8, 11, 12): PSCs occurred in old-aged individuals with significant male preponderance; Most patients had a smoking history; in 80% of cases the lesions were located in the upper lobe of the bilateral lung, especially in the peripheral lung; at diagnosis, most PSM presents as > 3cm mass; necrosis is frequent in lesions.

Serum tumor markers were variant in patients with PSC. In our study, serum CEA, SCCA, CYFRA 21-1, and NSE levels were increased in 12%, 24%, 36%, and 12% of all patients, respectively. The investigation by Ito et al. revealed that 63.6% of patients had an elevated CEA level and 55.6% had an elevated CYFRA 21-1 level (11). In another study of 24 PSC patients, only 2 (8.3%) presented with increased CEA levels, 2 (8.3%) with elevated CYFRA211 levels, 1 (4.2%) with increased SCCA level, and 1 (4.2%) with an increased NSE level (4). There are significant differences in tumor marker levels among PSC patients in different research groups, which may be related to the subtypes and NSCLC components of PSC. The variant limits the application of tumor markers in PSC diagnosis.

At diagnosis, although PSC is often large when detected, metastasis mainly involves the ipsilateral hilar and mediastinal lymph node, adjacent pleural (4, 12). Pleural invasion usually presents broad base infiltration, as shown in **Figure 1**. We have found a case of small intestine metastasis, which has been reported before (7, 9, 10). In other non-small cell lung cancers, gastrointestinal metastasis is extremely rare. In our case, intrapulmonary metastasis is not uncommon, which may be related to tumor vascular invasion. The study of Shishido et al. showed that more than half of the patients with pulmonary pleomorphic carcinoma had vascular invasion (12). The distant metastases of PSC were mainly in lymph nodes and bone. The brain is also a frequent site of metastasis (9).

From the previous and our studies, almost all PSCs revealed high glucose metabolism (4, 8, 9, 11, 12), and a study has shown that the glucose metabolism of PSCs is significantly higher than that of other NSCLCs (9). We found that there was a significant correlation of the SUVmax with the size of PSCs, the lesions with the longest diameter ≥ 5cm had higher SUVmax than those with the longest diameter < 5cm; This was different from previous reports (4). There may be many factors for the different conclusions. The expression of PD-L1 and gene mutations of PSC may be one of the factors contributing to this difference. Wu et al. ‘s research demonstrated the SUVmax of lesions with PD-L1 expression degrees ≥ 50% was significantly higher than that of lesions with PD-L1 expression degrees < 50%, and the SUVmax was significantly higher in the tumor with KRAS mutation than in those without KRAS mutations (4); ^18^F-FDG uptake was closely correlated with the expression of glucose transporters in varying amounts in PSC (13); Patients with high ^18^F-FDG uptake have the significantly higher mean scoring of Glut1, Glut3, VEGF positivity and number of microvessels (CD34) than those with low ^18^F-FDG uptake (8). Reliable conclusions require multifactor analysis of a larger sample. For patients with IVa and IVb stages in our study, SUVmax didn’t demonstrate a significant difference. Previous studies (4, 8) have revealed there was no significant difference in SUVmax among PSCs with different TNM stages. there was no significant difference in SUVmax of PSM between patients with OS >= 12 months and patients with OS < 12 months. A study have revealed that PSC’s SUVmax was associated with patient survival (9). However, in our study, there was no significant difference in SUVmax of PSM between patients with different OS. Rapicetta C et al. (14) found that P-stage, surgical radicality, vascular/lymphatic invasion but not SUVmax affected long-term survival in PSC. This maybe because patients in different study groups had different tumor stages. Further meta-analysis should be performed by combining the results from multiple studies to reach a reasonable conclusion.

TTF-1 is often expressed unexpectedly in a large number of cases of PSC. A multi-omics study (15) based on TTF-1 and P40 immunohistochemistry (IHC) established a new PSC typing method that was simpler and more economical than genotyping. they divided PSC patients into three subtypes (TTF-1 positive group, P40 positive group, and double negative group). The applicability of targeted therapy and immunotherapy in PSC patients was explored from the perspectives of mutation map, tumor mutation load, tumor immune cell infiltration, etc., to provide clues for the precise treatment of PSC. Our results showed that there was a significant difference in SUVmax between lesions with TTF-1 positive expression and those with TTF-1 negative expression. The SUVmax of the former was higher than that of the latter. PET/CT may also be helpful in classifying and guiding the treatment of PSC patients.

PSC is a kind of non-small cell lung cancer (NSCLC). Given the poor prognosis of PSC, early accurate identification of PSC is a critical need for the selection of an appropriate treatment regimen. However, the diagnosis of PSC with ^18^F-FDG PET/CT is also limited. In our group, 44% of lesions were considered for the possibility of PSC (2 confirmative PSC, 4 probable PSC, and 5 possible PSC) on ^18^F-FDG PET/CT. Shishido Y et al. reported only 3 cases diagnosed with PSC preoperatively in a cohort of 29 patients who had undergone PET/CT scan (12). An early histopathologic analysis is essential to not delay PSC diagnosis.

In this group, 5 patients underwent surgical treatment and 20 patients received pharmacological treatments, but survival analysis showed no significant difference in prognosis between surgical patients and non-surgical patients. One study (9) showed that surgical patients had a better prognosis than non-surgical patients. This should be because the stages of the two groups of patients with PSC were different.

Our investigation was a retrospective study conducted at a single center, the sample size was small due to the rarity of PSC, the vast majority of the participants were patients with stage IV PSC, selection bias was unavoidable.

In conclusion, we conducted a retrospective analysis of the clinical data and the ^18^F-FDG PET/CT findings for PSC. PSC is a kind of NSCLC, they have some clinical and imaging characteristics. However, other than size, the primary tumor SUVmax has no significant correlation with clinicopathological factors. In addition, we also found that SUVmax was associated with the expression of TTF-1 in PSCs. Although imaging diagnosis including PET/CT is still challenging, the location of the primary tumor, its large size, pleural invasion, and hypermetabolism are suggestive of PSC. Early histopathological diagnosis is necessary to help develop a reasonable regimen.

## Data availability statement

The original contributions presented in the study are included in the article/supplementary material. Further inquiries can be directed to the corresponding author.

## Ethics statement

Ethical approval for this investigation was obtained from the Research Ethics Committee of Jiangxi Provincial People’s Hospital. Written informed consent was obtained from the individuals or next of kin for the publication of any potentially identifiable images or data included in this article.

## Author contributions

Z-HL: Conceptualization, Funding acquisition, Writing – original draft, Writing – review & editing. X-YL: Formal Analysis, Investigation, Resources, Writing – original draft. W-LQ: Formal Analysis, Investigation, Resources, Writing – review & editing. QL: Formal Analysis, Investigation, Resources, Supervision, Writing – review & editing.

## References

[1] Travis WD Brambilla E Burke AP Marx A Nicholson AG. World Health Organization classification of tumours of the lung, pleura, thymus and heart. 4th ed. Lyon: WHO Press (2015). 10.1097/JTO.0000000000000663 26291007

[2] Sun L Dai J Chen Y Duan L He W Chen Q. Pulmonary sarcomatoid carcinoma: experience from SEER database and shanghai pulmonary hospital. Ann Thorac Surg (2020) 110:406–13. doi: 10.1016/j.athoracsur.2020.02.071 32268141

[3] Antunes MDS Hochhegger B Alves GRT Gazzoni FF Forte GC Andrade RGF. Postoperative computed tomography of insufflated lung specimens obtained by video-assisted thoracic surgery: detection and margin assessment of pulmonary nodules. Radiol Bras (2022) 55:151–5. doi: 10.1590/0100-3984.2021.0046 PMC9254709 35795601

[4] Wu X Huang Y Li Y Wang Q Wang H Jiang L. 18F-FDG PET/CT imaging in pulmonary sarcomatoid carcinoma and correlation with clinical and genetic findings. Ann Nucl Med (2019) 33:647–56. doi: 10.1007/s12149-019-01374-5 31165974

[5] Gao C Zou Q Liu H. Pulmonary sarcomatoid carcinoma with epiglottis and ileum metastasis detected by 18F-FDG PET/CT. Clin Nucl Med (2022) 47:231–3. doi: 10.1097/RLU.0000000000003936 34653056

[6] Ciarallo A Makis W Novales-Diaz JA Lisbona R. Sarcomatoid carcinoma (carcinosarcoma) of the lung mimics Malignant pleural mesothelioma on 18F-FDG PET/CT: a report of 2 cases. Clin Nucl Med (2012) 37:416–9. doi: 10.1097/RLU.0b013e31823ea47f 22391723

[7] Xie X Tu N Wang Q Cheng Z Han X Bu L. ^18^ F-FDG PET/CT imaging of small intestinal metastasis from pulmonary sarcomatoid carcinoma: Brief report and review of the literature. Thorac Cancer (2020) 11:2325–30. doi: 10.1111/1759-7714.13468 PMC7396377 32410331

[8] Kaira K Endo M Abe M Nakagawa K Ohde Y Okumura T. Biologic correlates of ^18^F-FDG uptake on PET in pulmonary pleomorphic carcinoma. Lung Cancer (2011) 71:144–50. doi: 10.1016/j.lungcan.2010.05.021 20646779

[9] Park JS Lee Y Han J Kim HK Choi YS Kim J. Clinicopathologic outcomes of curative resection for sarcomatoid carcinoma of the lung. Oncology (2011) 81:206–13. doi: 10.1159/000333095 22076573

[10] Koh H Chiyotani A Tokuda T Suzumura H Kamiishi N Takahashi H. Pleomorphic carcinoma showing rapid growth, multiple metastases, and intestinal perforation. Ann Thorac Cardiovasc Surg (2014) 20:669–73. doi: 10.5761/atcs.cr.13-00167 24492166

[11] Ito K Oizumi S Fukumoto S Harada M Ishida T Fujita Y. Clinical characteristics of pleomorphic carcinoma of the lung. Lung Cancer (2010) 68:204–10. doi: 10.1016/j.lungcan.2009.06.002 19577320

[12] Shishido Y Aoyama A Hara S Sato Y Tomii K Hamakawa H. Ringed fluorodeoxyglucose uptake predicted poor prognosis after resection of pulmonary pleomorphic carcinoma. J Cardiothorac Surg (2022) 17:47. doi: 10.1186/s13019-022-01799-6 35313902 PMC8935789

[13] De Geus-Oei LF van Krieken JH Aliredjo RP Krabbe PF Frielink C Verhagen AF. Biological correlates of FDG uptake in non-small cell lung cancer. Lung Cancer (2007) 55:79–87. doi: 10.1016/j.lungcan.2006.08.018 17046099

[14] Rapicetta C Lococo F Stefani A Rossi G Ricchetti T Filice A. Primary sarcomatoid carcinoma of the lung: radiometabolic ((18)F-FDG PET/CT) findings and correlation with clinico-pathological and survival results. Lung (2016) 194:653–7. doi: 10.1007/s00408-016-9904-1 27300448

[15] Yang Z Tian H Li L Li C Xu J Bie F. PSC subtyping based on TTF-1 and p40 expression reveals distinct molecular characteristics and therapeutic strategies. Int J Cancer (2022) 151:717–29. doi: 10.1002/ijc.34137 35612583

